# Cell cycle association and hypoxia regulation of excision repair cross complementation group 1 protein (ERCC1) in tumor cells of head and neck cancer

**DOI:** 10.1007/s13277-014-2001-2

**Published:** 2014-05-12

**Authors:** József Dudás, Volker H. Schartinger, Angela Romani, Gabriele Schweigl, Kristian Kordsmeyer, Patricia Irina Marta, Christoph Url, Florian Kral, Herbert Riechelmann

**Affiliations:** 1 Department of Otorhinolaryngology and Head and Neck Surgery, Medical University Innsbruck, Anichstrasse 35, 6020 Innsbruck, Austria; 2 Institute of Pathology, Medical University Innsbruck, Müllerstrasse 44, 6020 Innsbruck, Austria

**Keywords:** Genetic instability, Oropharynx, Hypopharynx, DNA repair, Homologue recombination

## Abstract

Excision repair cross complementation group 1 (ERCC1) is a key component of homologous recombination-based repair of interstrand DNA cross-links (ICLs). As a consequence, ERCC1 mediates resistance to mitomycin C (MMC) and platinum chemotherapeutic agents and may predict treatment failure. Clinical response to MMC or cisplatin (CDDP)-based radiochemotherapy (RCT) was assessed in 106 head and neck squamous cell carcinoma (HNSCC) patients and correlated with cell nuclear immunoreactivity of the mouse monoclonal (clone: 8 F1) ERCC1 antibody in tumor tissue samples. BEAS-2B epithelial and Detroit 562 pharyngeal squamous carcinoma cells were treated with CDDP, MMC, and 5-fluorouracil (5-FU) at 50 % growth inhibitory (IC-50) concentrations. ERCC1 protein synthesis was compared with cell cycle distribution using combined immunocytochemistry and flow cytometry. ERCC1 messenger RNA (mRNA) and protein expression was investigated in normoxic and hypoxic conditions in Detroit 562 cells. Clinically, the nonresponder revealed significantly lower HNSCC tissue ERCC1 immunoreactivity than the responder (*p* = 0.0064) or control normal mucosa, which led to further mechanistic investigations. In vitro, control cells and cells treated with cytotoxic agents showed increasing ERCC1 levels from the G1 through S and G2 phases of the cell cycle. In CDDP-treated cells, ERCC1 mRNA and protein expression increased. Under hypoxic conditions, ERCC1 gene expression significantly decreased. Although ERCC1^+^ cells show increased chemoresistance, they might be particularly radiosensitive, representing G2 cell cycle phase and less hypoxic. ERCC1 expression might be indirectly related with some conditions important for RCT treatment, but it is not a clear predictor for its failure in HNSCC patients.

**Electronic supplementary material:**

The online version of this article (doi:10.1007/s13277-014-2001-2) contains supplementary material, which is available to authorized users.

## Introduction

The Xeroderma pigmentosum group F protein (XPF) and excision repair cross complementation group 1 (ERCC1) exist as a heterodimer, which is a structure-specific nuclease necessary for the repair of interstrand DNA cross-links (ICLs) [1]. Environmental carcinogens contained in air pollution, such as polycyclic aromatic hydrocarbons, aromatic amines, or N-nitroso compounds, predominantly form DNA adducts and also generate ICLs [2]. ICLs are an extremely toxic class of DNA damage incurred also during cancer chemotherapy. They covalently tether both strands of duplex DNA, preventing the strand unwinding that is essential for polymerase access. In consequence, ICLs induce double strand break (DSB) formation during cell cycle progression into S phase. Previously published evidence demonstrated that ERCC1 is required for the resolution of cross-link-induced DSB, which leads to the subsequent repair of cytotoxic intermediates by homologous recombination [3]. ERCC1-deficient mice show severe growth retardation associated with premature replicative senescence leading to liver failure and death at 4 weeks of age [4]. ERCC1 knocked down cells were delayed in their cell cycle and became multinucleated. These data have suggested that ERCC1 might also play a role in mitotic progression, which may be critical during development [5]. The ERCC1/XPF complex has been proposed to play a role in mitotic recombination as well as in nucleotide exchange repair [4]. If cells that cannot repair their damaged DNA proceed through either replication or binucleation (which involves a round of replication followed by an acytokinetic cell division), this could lead to the fixation of mutations and the creation of chromosomal aberrations [4].

Current data on the prognostic and predictive value of ERCC1 tumor immunoreactivity are inconsistent. A previous study has reported that low ERCC1 expression is associated with genomic instability and higher risk of cancer [6, 7]. Increased genetic instability was related with increased recurrence risk [8] and worse long-term outcome of head and neck squamous cell carcinoma (HNSCC). In fact, chemotherapeutic agents such as cisplatin, cis-dichloro-diamine-platinum (CDDP) also induce ICLs, and apparently, high immunohistochemical detection of ERCC1 might be associated with CDDP resistance. Referring to a previous study, patients with low expression of ERCC1 had significantly higher 3-year progression-free and overall survival rates [9]. In accordance, patients with low ERCC1 expression are more likely to benefit from CDDP induction chemotherapy than patients with high ERCC1 expression [10, 11]. In contrast, Koh et al. reported that ERCC1 tumor immunoreactivity had no effect on chemotherapy response in patients with oropharyngeal, hypopharyngeal, and laryngeal cancer treated with CDDP containing induction chemotherapy [12]. Moreover, De Castro et al. recently reported that high ERCC1 expression seems to be associated with better overall survival rates in HNSCC patients submitted to adjuvant cisplatin-based chemoradiation [13]. Confirming these data, studies done in two other tobacco-related cancers, namely nonsmall cell lung cancer and pancreatic adenocarcinoma, treated by surgery alone, suggest that ERCC1 positivity may implicate a favorable prognosis [14]. In a recent publication, Bisof and coworkers mentioned that studies on the association of ERCC1 expression and survival in head and neck carcinoma patients treated with definitive or adjuvant radiation therapy yielded conflicting results [15]. In fact, Bisof and coauthors have published that ERCC1 expression had no impact on overall survival in head and neck carcinoma patients treated with definitive radiotherapy (DR) or adjuvant radiotherapy (AR) [15]. Similarly, Johung and coauthors published that ERCC1 expression did not predict radiotherapy resistance in laryngeal cancer [16]. In all these studies, ERCC1 gene expression was assessed employing immunohistochemistry with the same antibody clone (8 F1).

Pretreatment immunohistochemical analysis of ERCC1 protein representation in formalin-fixed paraffin-embedded (FFPE) tumor biopsies obtained during diagnostic endoscopy was established at our department on a weekly routine basis. Similar to the results of De Castro et al. [13], we found a positive relation between complete response after primary concomitant radiochemotherapy (RCT) (5-fluorouracil (5-FU)/mitomycin C (MMC) or 5-FU/CDDP) and high representation of ERCC1 in tumor cell nuclei. Since there was no mechanistic explanation for this result, we initiated some in vitro investigations. In detail, we compared two ERCC1 antibodies in relation to XPF expression and examined ERCC1 expression in vitro under normoxic and hypoxic conditions in response to MMC and CDDP in relation to cell cycle phases.

## Materials and methods

### Patient characteristics

The procedures followed were in accordance with the ethical standards of the committee on human experimentation of the institution or in accord with the Helsinki Declaration of 1975 as revised in 1983. Permission was obtained from the local ethics committee to collect pretreatment biopsy samples for paraffin embedding, sectioning, and immunohistochemical analysis (Reference Number: UN4428 303/4.14). Patients with locally advanced unresectable squamous cell carcinomas of the head and neck region treated between April 2008 and October 2013 with primary RCT at the Department of Otorhinolaryngology—Head and Neck Surgery, Medical University Innsbruck, were consecutively included. Pretreatment tumor samples were obtained during diagnostic panendoscopy. Tumor biopsies were immunohistochemically stained with the ERCC1 antibody 8 F1 from Acris (Herford, Germany). The proportion of ERCC1^+^ cells and nuclei in tumor cell nests was counted in 10 high power fields. All patients received primary RCT. Treatment response was assessed 6–8 weeks after the end of treatment and grouped into either complete response or treatment failure, because incomplete responses were associated with poor survival. Clinical data are summarized in Supplementary Table 1. Twenty-six normal mucosa tissue specimens were derived from uvulopalatopharyngoplasties (UPPP) and other nontumor samples.

### Treatment and clinical evaluation of treatment response

Patients underwent treatment upon multidisciplinary tumor board recommendation. The standard treatment for advanced HNSCC not suited for surgical treatment was primary radiochemotherapy (pRCT). Standard pRCT consisted of percutaneous normofractionated computer tomography CT-supported 3D-planned radiotherapy (RT) with 70–72 Gy, combined with CDDP or mitomycin C and 5-fluorouracil according to common protocols [17–19]. In patients not eligible for chemotherapy, RT with concomitant weekly cetuximab or—in case of cetuximab intolerance—a hyperfractionated RT protocol was administered [20, 21] (detailed in Supplementary Table 1). A complete diagnostic workup including biopsies from the initial tumor regions was performed 8 to 12 weeks following end of treatment. Moreover, normal mucosa samples of 26 nontumor patients were included. With regard to the study aim, these patients had no relevant mucosal abnormalities.

### ERCC1 immunohistochemistry

Five-micrometer-thick paraffin sections were dewaxed and antigen retrieval was performed in a Discovery^TM^ automated staining system (Ventana, Tucson, AZ, USA). ERCC1 antibodies were added to the sections by manual titration (mouse monoclonal clone 8 F1 Acris, 1:100 dilution), and the staining was completed by the Discovery^TM^ automated staining system using universal secondary antibody solution, hematoxylin counterstaining, and the DAB MAP Kit (all Ventana products). All sections were stained with control mouse and rabbit immunoglobulins, using the same highest concentration as for the primary antibodies, and these controls were not reactive [22]. The immunohistochemical reactions were observed independently by two blinded observers, who collected 10 representative tumor cell nests from each specimen [22], or 10 random areas of normal mucosa (UPPP samples). These regions were analyzed on an Olympus BX50 microscope (Tokyo, Japan) and on the TissueFaxs system (Tissuegnostic, Vienna, Austria). The whole number of cells and the number of immunopositive cells were counted in 10 high power fields (HPF). The staining index (percent of positive cells) was calculated as the average ratio between the positive cells and whole cell number [22]. Correspondingly, the nuclear staining index was calculated for cell nuclei only.

### Comparison of the immunohistochemical reaction of 8 F1 and FL-297 ERCC1 antibodies and of a XPF antibody on a random sample of patients

Paraffin sections of 18 HNSCC specimens (Supplementary Table 2) were used to compare the immunohistochemical reactivity of two ERCC1 antibodies: mouse monoclonal, clone 8 F1 from Acris (1:100 dilution), and rabbit polyclonal, FL-297 from Santa Cruz Biotechnology (Santa Cruz, CA, USA, 1:250 dilution) as previously described and suggested by Niedernhofer and coworkers [23–25]. For immunohistochemistry of XPF, the mouse monoclonal antibody SPM228 (1:1,000; Abcam, Cambridge, MA) was used [26].

### ERCC1 gene expression under normoxic and hypoxic cell culture conditions

BEAS-2B immortalized noncancer bronchial epithelial cells [27] and Detroit 562 metastatic pharyngeal squamous cell carcinoma cells were used [28]. Both cell lines were of commercial origin, and their culture conditions have been published before [28]. In some western blot experiments, SCC-25 oral squamous cell carcinoma cells were used.

Hypoxic cell culture conditions were performed in a Memmert INCO 108 incubator (Schwabach, Germany), which provides controlled 1 % O_2_. Detroit 562 cells were plated at 5 × 10^4^/ml in six-well plates at 3 ml/well. After an initial 48-h culture period, one plate was used as “control zero” for RNA isolation and real-time PCR. The further plates were used for RNA isolation and protein fractionation in three repeats. Cells were cultured after the initial 48-h normoxic culture for 48–96 h normoxic, or for 48–72 h in hypoxic conditions, and also combined, 48 h hypoxic followed by 24 or 48 h normoxic conditions. These culture conditions were based on a published reference [29]. Gene expression of ERCC1 was analyzed related to the “control zero” condition.

For protein synthesis analysis, cells were plated at 5 × 10^4^/ml in six-well plates (3 ml cell suspension/well), grown for 4 days, and on the fifth day, cells in one well were scraped into 500 μl extraction buffer followed by nonnuclear and 0.35 M NaCl nuclear protein fractionation as described previously [30]. Also, cells in hypoxic or normoxic or combined culture were used for nuclear protein fractionation. Nuclear and nonnuclear protein fractions were subjected to protein concentration measurement, and 10 μg proteins were used for western blots with antibodies of SPM228 (XPF, in 1:1,000 dilution), 8 F1 (ERCC1, mouse monoclonal, in 1:250 dilution), and FL-297 (ERCC1, rabbit polyclonal, in 1:200 dilution). Blots were processed in a western blot protocol previously detailed [31].

### ERCC1 gene expression under chemotherapy (IC-50 dose)

BEAS-2B and Detroit 562 cells were plated at 5 × 10^4^/ml in 96-well plates, 100 μl/well, were cultured for 48 h, and were treated with 0.1–100 μmol/l CDDP [32] or MMC [33] or 5-FU [34] for 48 h in complete medium (with medium replacement after 24 h), according to previous publications [33–35]. After 48 h of treatment, the medium was replaced with a fresh one, and after 24 h, the (3-(4,5-dimethylthiazol-2-yl)-2,5-diphenyltetrazoliumbromide (MTT) assay was performed as described previously [35]. The formazan crystals were dissolved in 10 % sodium dodecyl sulfate- and 10 mM HCl-containing solution, and the absorption was measured at 550 nm using an Anthos 2010 ELISA reader (Salzburg, Austria). The absorption values of cell-free blank wells were removed from the absorption values of cell-containing wells, and the average absorption value of untreated cells was considered as 100 % growth capacity. The absorption values of the treated cells were related to the controls as percentage of growth capacity. The relationship between growth capacity and treatment drug concentrations was analyzed by regression using nonlinear dose-response fitting methods of GraphPad Prism 4.03 (GraphPad Software Inc, San Diego, CA, USA), and concentrations were determined, which cause 50 % growth inhibition compared to untreated cells (IC-50).

ERCC1 gene expression at the messenger RNA (mRNA) level has been determined after treatment with IC-50 doses of CDDP, MMC, and 5-FU. BEAS-2B and Detroit 562 cells were plated at 5 × 10^4^/ml in 10 cm cell culture dishes, 10 ml/dish, and were then cultured and treated as described by the IC-50 determination with IC-50 concentrations of CDDP or MMC or 5-FU for 48 h in complete medium (with medium replacement after 24 h). After 48 h of treatment, the medium was replaced with a fresh one, and after 24 h of culture in complete medium, the cells were used for immunocytometry [36] or for RNA isolation [37–39]. For RNA isolation, the same cells and treatments were performed as for immunocytometry. RNA was reverse-transcribed as published before [31, 38], and gene expression levels of ERCC1 were determined according to previous reports [31, 38–40]. All used human PCR primers were published previously: ERCC1 [41]; β-actin [42], which was used as a housekeeping gene, was not regulated in control and treated samples and also not in hypoxic cultures. The relative gene expression was calculated as previously reported [42].

### Cell cycle dependence of ERCC1 protein synthesis

After the above-detailed cell culture and treatments, cells were trypsinized and suspended in 750 μl ice-cold phosphate buffered saline (PBS)/MgCl_2_ (PBS containing 2 mM MgCl_2_) / 10^6^ cells, and 250 μl cold 1 % BD-Cellfix (Becton Dickinson, Vienna, Austria) was added under continuous vortexing [36]. This mixture was incubated on ice for 1 h, and the cells were centrifuged at 1,200 rpm for 5 min at 4 °C, followed by 15 min incubation in 0.2 % Tween-20/PBS/10^6^ cells at 37 °C. The cells were centrifuged at 1,200 rpm for 5 min at 4 °C again and then incubated in 5 % bovine serum albumin in PBS/MgCl_2_ at room temperature for 20 min followed by centrifugation at 1,200 rpm for 5 min at 4 °C. The cells were resuspended in 1 % bovine serum albumin in PBS/MgCl_2_ and were incubated either with isotype-specific control mouse IgG2b, or with ERCC1 (8 F1, Acris) antibody at 100 times dilution at room temperature for 90 min followed by centrifugation at 1,200 rpm for 5 min at 4 °C. The immunocytochemical reaction was detected by 100 times diluted anti-mouse Alexa 488 (Jackson ImmunoResearch, Newmarket, Suffolk, UK) incubated in 1 % bovine serum albumin in PBS/MgCl_2_ at room temperature for 30 min. Subsequently, DNA was stained with propidium iodide for cell cycle analysis as published before [35]. The double-stained cells were flow cytometrically examined on a Coulter Epics XL-MCL (Beckman Coulter, Brea, CA, USA). In addition to the cells that reacted through the whole procedure, the following controls were used for setting up the flow cytometry conditions and for compensation: cells reacted with whole antibodies but without propidium iodide and anti-mouse Alexa 488, cells immunoreacted with isotype-specific control mouse IgG2b and DNA-stained with propidium iodide, and cells reacted with ERCC1 (8 F1, Acris) antibody but DNA not stained with propidium iodide. Frequency diagrams (histograms; fluorescence intensity on the *X*-axis, events on the *Y*-axis, or fluorescence intensity on the *X*-axis, events related to all events on the *Y*-axis) and scattergrams were recorded for Alexa 488 at the fluorescence-1 (FL-1), and for propidium iodide at the fluorescence-1 (FL-3) channel of a Beckman Coulter XL-MCL. Doublets and adhered cells were removed from the DNA analysis using FL3 signal height/signal area graphs.

### Statistical analysis

Each experiment was performed in four independent sets containing four biological repeats/set. The raw experimental results were tested for normal distribution by D’Agostino and Pearson omnibus normality test using the GraphPad Prism 4.03 (GraphPad Software Inc, San Diego, CA, USA). Average values were compared by nonparametric tests (Mann-Whitney) or by Student’s *t* tests depending on the distribution of the data. Logistic analysis was performed using MedCalc 12.4 (Ostend, Belgium). Correlation analysis of the staining index of ERCC1 and XPF antibodies was performed by the “Pearson *r*” method using GraphPad Prism 4.03.

## Results

### Low ERCC1 expression was associated with treatment failure after primary RCT

**Fig. 1:**
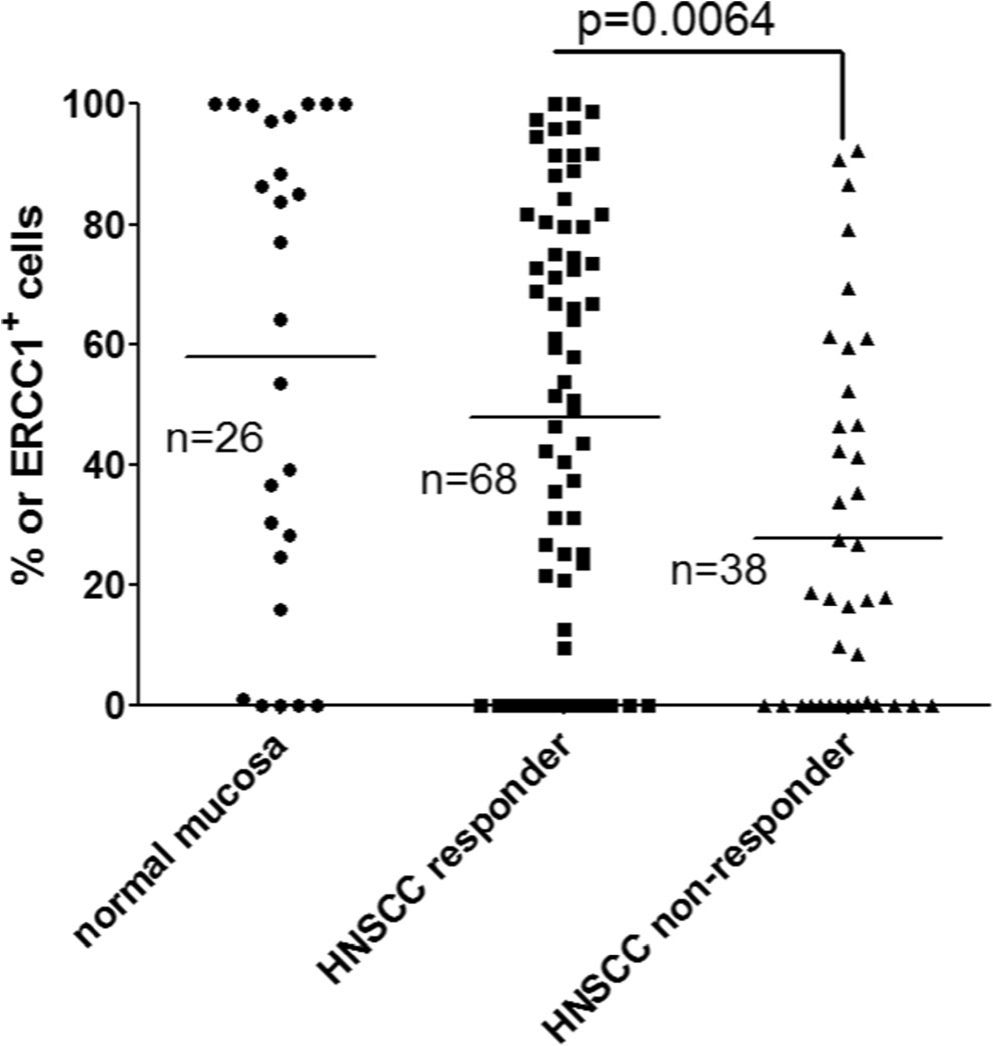
Twenty-six normal mucosa samples and 106 HNSCC tumor tissue samples have been stained with 8 F1 ERCC1 antibody, and the percentage of stained cells in tumor cell nests or in high power fields of normal mucosa has been evaluated. There was no significant difference between the staining representation of normal mucosa and therapy responder HNSCC, but the therapy nonresponder HNSCC patients have shown significantly lower (*p* = 0.0064) ERCC1 representation compared to the normal mucosa and responder patients

During the study period, 106 patients with unresectable HNSCC treated with primary concomitant RCT were included. Of these, 68 responded completely, while 38 failed. Tissue samples of 26 patients without tumor containing normal mucosa served as controls. ERCC1 staining indices between these three groups differed significantly (*p* < 0.01). Particularly, tumor specimens of treatment failures revealed significantly lower ERCC1 expression (staining index 28 ± 30 %; mean ± SD) than specimens from controls (58 ± 40 %; *p* = 0.0064), while specimens from treatment responders (48 ± 35 %) did not (*p* > 0.05) (Fig. 1). Taking different sites of HNSCC into consideration, this tendency has been seen in the oral cavity, oropharynx, hypopharynx, and larynx, but the highest difference between therapy responder and nonresponder patients was found in the hypopharynx (Supplementary Material 3).

These results did not comply with some recent publications suggesting an association of treatment failures with high ERCC1 expression [10, 11]. This brought us to reevaluate the role of ERCC1 on a mechanistic level. In the first step, we wanted to rule out that simple unspecificity of the antibody clone 8 F1 might bias these results [23, 24, 26].

### Comparison of 8 F1 and FL-297 ERCC1 antibodies

**Fig. 2:**
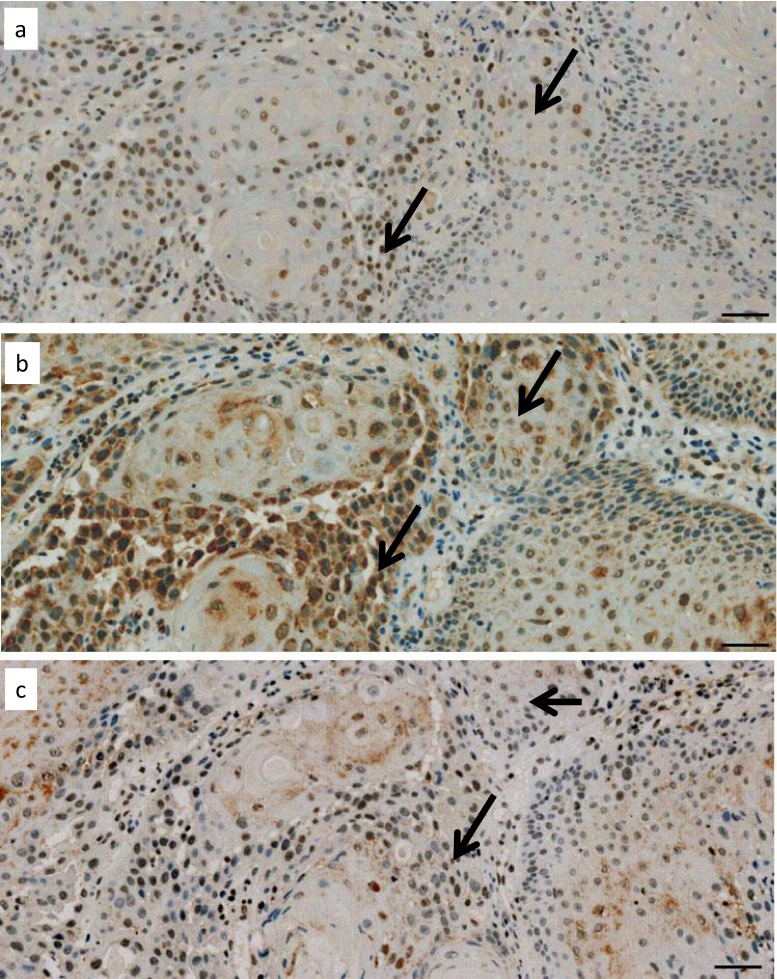
Immunohistochemical analysis of ERCC1 using antibodies 8 F1 (**a**) and FL-297 (**b**) and of XPF using SPM228 (**c**) in serial sections of nasopharynx squamous cell carcinoma. The 8 F1 ERCC1 and the SPM228 XPF antibodies show cell nuclear reaction in comparable areas of serial sections, while the FL-297 ERCC1 antibody shows mainly perinuclear and cytoplasmic reaction in comparable areas of serial sections. The reaction of FL-297 antibody is more extended than the one of the other two, which fit each other. *Bars* = 50 μm

Recently, the specificity of the most frequently used ERCC1 antibody clone 8 F1 was doubted by Niedernhofer and coworkers and another ERCC1 antibody, FL-297, was favored [23, 24, 43]. In a random sample of 18 tumor specimens, we compared the immunohistochemical reaction products to 8 F1 (ERCC1), FL-297 (ERCC1), and to the XPF antibody SPM228, the partner of ERCC1 in dimer formation. For ERCC1 as a DNA excision repair enzyme, nuclear presentation was expected. The 8 F1 antibody revealed relative homogenous cell nuclear staining in HNSCC specimens (Fig. 2a, arrows), while heterogeneous perinuclear staining and cytoplasmic reactions were observed with the FL-297 antibody (Fig. 2b, arrows). The SPM228 antibody homogenously stained the cell nuclei (Fig. 2c, arrows).

Nuclear staining indices for 8 F1 (ERCC1) and SPM228 (XPF) correlated significantly (*r* = 0.51; *p* = 0.021) (Table 1, Supplementary File 4), while not for SPM228 and FL-297 (*r* = 0.031; *p* = 0.904). Moreover, the ERCC1 staining with FL-297 and 8 F1 did not correlate (*r* = 0.159; *p* = 0.53) (Table 1, Supplementary File 4).

**Table 1:** Correlation analysis of ERCC1 and XPF cell nuclear representation using antibodies of 8 F1 and FL-297 for ERCC1 and SPM228 for XPF

Antibody evaluated on the *X*-axis	Antibody evaluated on the *Y*-axis	Distribution of the data on the *X*- and *Y*-axes	Pearson *r*	*p* value	Represented	Comment
8 F1 (ERCC1)	FL-297 (ERCC1)	Normal distribution	0.159	0.5276	Supplementary File 4	No correlation
8 F1 (ERCC1)	SPM228 (XPF)	Normal distribution	0.510	0.021	Supplementary File 4	Significant correlation
FL-297 (ERCC1)	SPM228 (XPF)	Normal distribution	0.031	0.904	Supplementary File 4	No correlation

Taken together, ERCC1 detected by the 8 F1 antibody clone revealed the expected nuclear representation and correlated with its functional partner XPF, while polyclonal FL-297 localized perinuclear and cytoplasmic and its staining distribution did not correlate with that of XPF. We interpret these data that the required immunohistochemical specificity of the 8 F1 clone for ERCC1 detection is sufficient and that the unexpected low ERCC1 expression in HNSCC treatment failures was not due to unspecific staining of the antibody employed. This conclusion was supported by western blot investigations in cell lines (see below).

### Specificity of the 8 F1 antibody clone in squamous cell carcinoma cell lines

For western blot analyses, oral SCC-25 [38] and pharyngeal Detroit 562 [44] squamous cell carcinoma cells and noncancer virally immortalized bronchial epithelial BEAS-2B cells [27] were employed. ERCC1 gene expression was assessed using real-time RT-PCR. In all of the investigated cells, significant ERCC1 gene expression was detected with Ct values of 25–28.

**Fig. 3:**
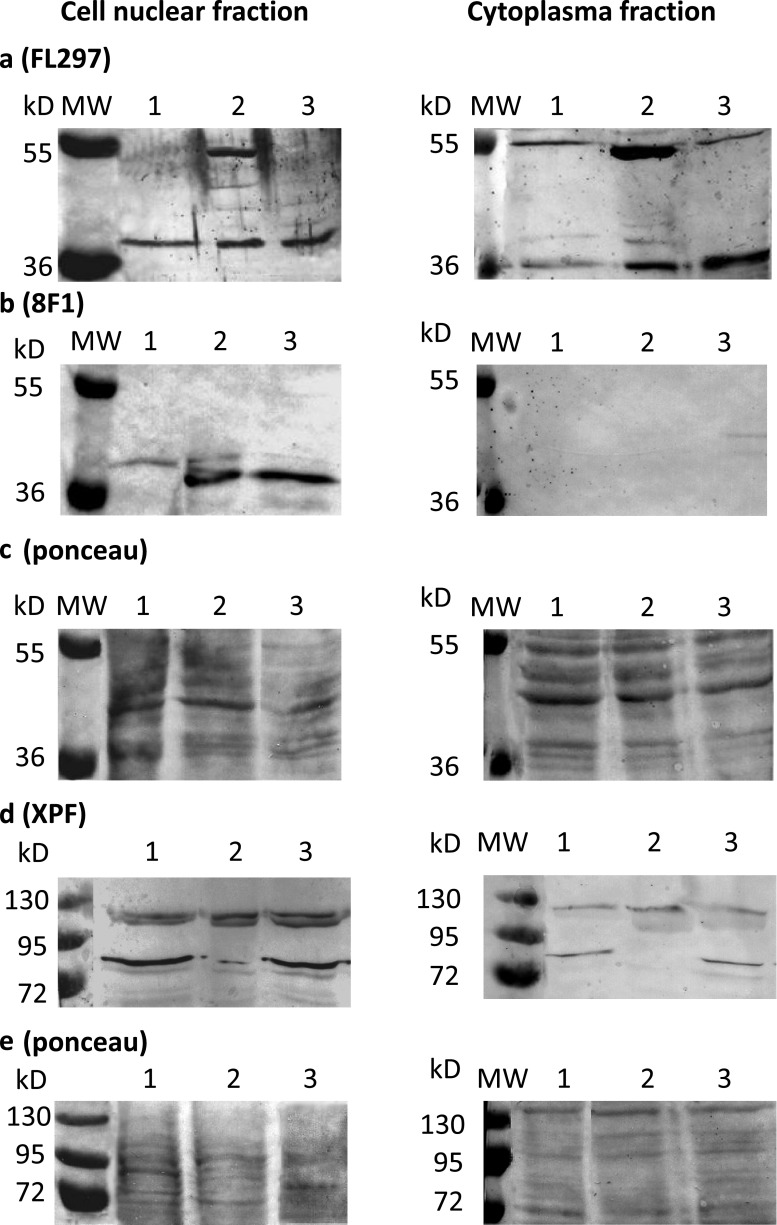
Western blot of FL-297 (**a**) and 8 F1 (**b**) anti-ERCC1 antibodies and of SPM228 (**d**) anti-XPF antibody in SCC-25 (*lane 1*) and Detroit 562 (*lane 2*) squamous cell carcinoma cell lines and in not transformed BEAS-2B bronchial epithelial cells (*lane 3*) using cell nuclear (*left panels*) and cytoplasm (*right panels*) fractions. Ponceau-stained whole protein detection was used as loading control (**c**, **e**)

Cell nuclear (histone-free) and cytoplasm extracts were separated by centrifugation and utilization of NP-40 [30] for western blot analyses. ERCC1 was expected at 37 kDa and XPF at approximately 116 kDa [25]. The rabbit polyclonal FL-297 antibody raised against ERCC1 recognized a band at 37 kDa in all examined cell lines and an additional band in Detroit 562 cells at 55 kDa (Fig. 3a). This antibody also recognized the same bands in the cytoplasm extracts. Based on the available data [25], only the 37-kDa band can be considered as specific.

The mouse monoclonal 8 F1 antibody raised against ERCC1 recognized a band at 37 kDa in Detroit 562 and BEAS-2B cells in the cell nuclear extract and did not show any bands in cytoplasmic extracts (Fig. 3b). The SPM228 mouse monoclonal antibody raised against XPF recognized the full XPF protein at 116 kDa and its known degradation derivative [25] in all examined cell lines both in the cell nuclear and cytoplasm extracts (Fig. 3d). Based on these results, we assume that the specificity of the 8 F1 antibody against ERCC1 was higher than the one of FL-297, supporting the results of the immunohistochemical investigations in patient tissues.

Considering the protein and mRNA expression data, Detroit 562 and BEAS-2B cells were chosen for further tests, because they reliably revealed the characteristic 37-kDa ERCC1 band using several antibodies (Fig. 3a, b). In these cell lines, ERCC1 expression has been also confirmed at the mRNA level (not shown). Moreover, for ERCC1 protein detection, the 8 F1 antibody was considered most suitable, because it specifically recognized the ERCC1 protein only at 37 kDa, and the reaction products were confined to the nucleus (Fig. 3b).

### ERCC1 gene expression at IC-50 of CDDP, MMC, and 5-FU

In the next step, we investigated the relation of ERCC1^+^ and ERCC1^−^ cells with the outcome of the experimental treatment with chemotherapeutic drugs. For this purpose, the IC-50 values had to be determined. Using a tetrazolium-based colorimetric assay (3-[4,5-dimethylthiazol-2-yl]-2,5-diphenyltetrazoliumbromide (MTT) assay), the IC-50 (drug concentration inducing a 50 % growth reduction in comparison to the control untreated cells) was determined in Detroit 562 and BEAS-2B cells for CDDP, MMC, and 5-FU. Drug concentration ranges for the treatment series were based on relevant publications [32–34, 45]. For Detroit 562 cells, the IC-50 value for CDDP was 4.84 μM, for MMC 0.5 μM, and for 5-FU 6.32 μM. For BEAS-2B cells, the IC-50 value for CDDP was 4.87 μM, for MMC 0.43 μM, and for 5-FU 95.08 μM.

After IC-50 treatment with CDDP, the relative protein levels (whole fluorescence intensity at FL-1 channel in all events together) increased to 138.08 ± 28.75 % (control 100 %) in Detroit 562 cells and to 115.53 ± 17.01 % in BEAS-2B cells. After MMC IC-50 treatment, the relative protein levels changed to 105.89 ± 13.82 % (control 100 %) in Detroit 562 cells and to 103.98 ± 5.63 % in BEAS-2B cells. After 5-FU IC-50 treatment, the relative protein levels changed to 122.49 ± 2.95 % (control 100 %) in Detroit 562 cells and to 91.88 ± 11.61 % in BEAS-2B cells.

For comparison, the ERCC1 gene expression was also investigated at the mRNA level following the same treatment conditions. CDDP treatment resulted 2.43 times and 1.43 times upregulation of gene expression (*p* < 0.05) in Detroit 562 and BEAS-2B cells, respectively (control 1.00), whereas MMC treatment did not influence the ERCC1 mRNA levels significantly in either Detroit 562 or BEAS-2B cells (*p* > 0.05). Treatment with 5-FU at IC-50 resulted in a significant (*p* < 10^−4^) 2.37 times increase of ERCC1 gene expression in Detroit 562 cells, while in BEAS-2B cells, the ERCC1 gene expression tended to decrease (*p* = 0.051) to 80 % (0.8 times) of the control level.

### Cell cycle relation of ERCC1 in control and treated cells

As a possible reason for the favorable response of HNSCC patients expressing high levels of ERCC1, we hypothesized that high ERCC1 staining represents particularly radiosensitive cells which are in the G2 phase of the cell cycle [46]. To test this hypothesis, we established an ERCC1 immunocytochemical analysis combined with propidium iodide DNA staining in flow cytometry [36, 47]. Then, we used this method on untreated and IC-50-treated Detroit 562 and BEAS-2B cells to assess cell cycle dependence of ERCC1 expression under these conditions.

In untreated Detroit 562 and BEAS-2B cells, the cell cycle distribution (Table 2) resembled a normal proliferating cell culture. Cells containing lower DNA content than diploid (sub-G1 phase) were infrequent. In both cell lines, cells in the G1 phase occurred most frequently. The cells with DNA content between diploid and tetraploid were identified as S phase, and cells with tetraploid DNA content were identified as G2 (G2/M) phase. S and G2 phases were comparable in untreated cultures of Detroit 562 and BEAS-2B cells (Table 2). After CDDP treatment at IC-50 for 48 h, followed by 1 day without treatment, Detroit 562 cells accumulated in the S and G2 phases and the representation of sub-G1 cells had also increased, which is clearly recognizable in Table 2. The effects of this treatment on BEAS-2B cells were quite similar. After MMC treatment at IC-50 for 48 h, followed by 1 day without treatment, both Detroit 562 and BEAS-2B cells revealed histograms similar to those after CDDP treatment, with increases of cell numbers in the sub-G1 and increase or maintenance of S and G2 phases (in Table 2, it is more evident for Detroit 562 cells). The most characteristic is the decrease of the representation of the G1 phase (Table 2). After 5-FU treatment at IC-50 concentration for 48 h, followed by 1 day without treatment, Detroit 562 cells accumulated in the G1-S phase, and the representation of sub-G1 cells had also increased (Table 2). The 48-h IC-50 (as determined by Detroit 562 cells) 5-FU treatment of BEAS-2B cells followed by 1 day without treatment has led to S phase block (nearly 50 % of all events in the S phase, Table 2), showing this as the main cell cycle phase, but the representation of sub-G1 cells has also increased (Table 2).

**Table 2:** Cell cycle distribution of Detroit 562 and BEAS-2B cells in untreated and in IC-50 CDDP-, MMC-, and 5-FU-treated conditions: mean ± SD, results of five repeats. Not always were all events recognized in any of the sub-G1, G1, S, or G2 phases, and the percent values do not necessarily give together 100 %

	Untreated	CDDP	MMC	5-FU
Detroit 562				
Sub-G1	5.2 ± 1.9	20.5 ± 8.8	24.1 ± 9.4	19.8 ± 3.2
G1	45.2 ± 5.0	14.5 ± 5.8	13.1 ± 4.6	28.2 ± 6.9
S	20.1 ± 3.3	23.3 ± 3.4	27.6 ± 4.3	23.4 ± 6.0
G2	22.8 ± 4.4	32.0 ± 10.7	28.2 ± 17.8	25.7 ± 1.8
BEAS-2B				
Sub-G1	3.2 ± 1.6	22.3 ± 0.7	33.8 ± 5.8	25.1 ± 3.9
G1	42.8 ± 3.9	17.1 ± 1.0	15.0 ± 1.2	14.2 ± 1.5
S	17.0 ± 2.5	20.5 ± 1.3	15.2 ± 2.9	45.9 ± 1.4
G2	26.9 ± 1.3	22.2 ± 3.0	26.9 ± 6.2	7.8 ± 1.7

**Fig. 4:**
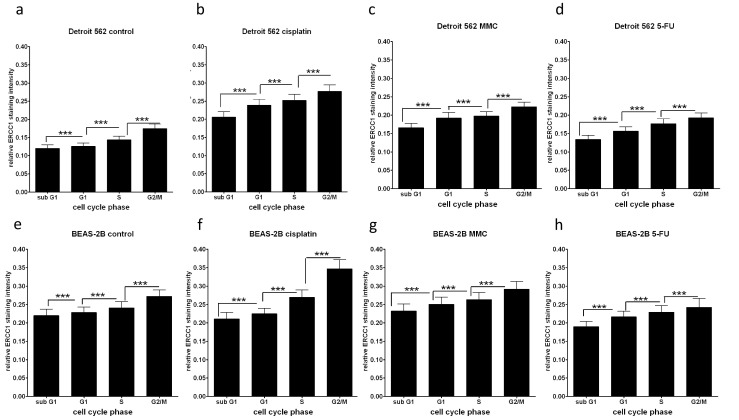
ERCC1-related fluorescence intensities in Detroit 562 (**a**–**d**) and BEAS-2B (**e**–**h**) cells gained with the ERCC1-specific immunocytochemical reaction. The “proportional events” (*p*_e_) (events corresponding to the ERCC1 fluorescence levels related to all events at the same cell cycle phase) were multiplied with the arbitrary fluorescence units (relative fluorescence intensity: *I*_f_) measured at the FL1 channel in all cell cycle phases to get the “cell-cycle-related ERCC1 immunocytochemical values” (*p*_e_ * *I*_f_). These data represent both the relative distribution of the cells at cell cycle phases and the ERCC1 staining intensity of the cells within the cell cycle phases. In fact, the graphs represented show the cell cycle distribution of ERCC1 protein. Cell cycle-related ERCC1 staining intensity in untreated (**a**, **e**) and CDDP- (**b**, **f**), MMC- (**c**, **g**), and 5-FU-treated (**d**, **h**) conditions. ***p < 0.001

Using the histograms measured in flow cytometry, cells in the sub-G1, G1, S, and G2 phases were gated and were further analyzed for fluorescence intensity measured with the ERCC1-specific immunocytochemical reaction and graphed on “FL1 − fluorescence intensity − relative cell numbers” histograms. The “relative events” (events corresponding to the ERCC1-related fluorescence levels related to all events at the same cell cycle phase) were multiplied with the arbitrary fluorescence units measured at FL1 channel in all cell cycle phases to get the “cell-cycle-related ERCC1 immunocytochemical values.” As seen in Fig. 4, in untreated and in CDDP-, MMC-, and 5-FU-treated Detroit 562 (Fig. 4a–d) and BEAS-2B (Fig. 4e–h) cells, the ERCC1 immunocytochemical values significantly increased from sub-G1, over G1 and S up to G2/M phases of the cell cycle. This tendency was observed independently from the chemotherapeutic agent employed.

CDDP treatment increased the ERCC1-related immunofluorescence levels (from 0.13–0.20 to 0.20–0.30) in both Detroit 562 and BEAS-2B cells (Fig. 4b, f), especially in the G2/M phase. These data indicate that ERCC1 is most represented in the radiosensitive G2/M phase, and CDDP, but not MMC, induces additional ERCC1 expression during treatment.

### Hypoxia significantly downregulates ERCC1 in Detroit 562 tumor cells

In a recent publication, Bindra et al. suggested a novel mechanism of genetic instability in the tumor microenvironment mediated by hypoxia-induced suppression of the homologous recombination pathway in cancer cells [29]. The ERCC1-XPF dimer is a component of the complex machinery in homologous recombination [3]. We were therefore interested in the effect of hypoxia on ERCC1 expression.

**Fig. 5:**
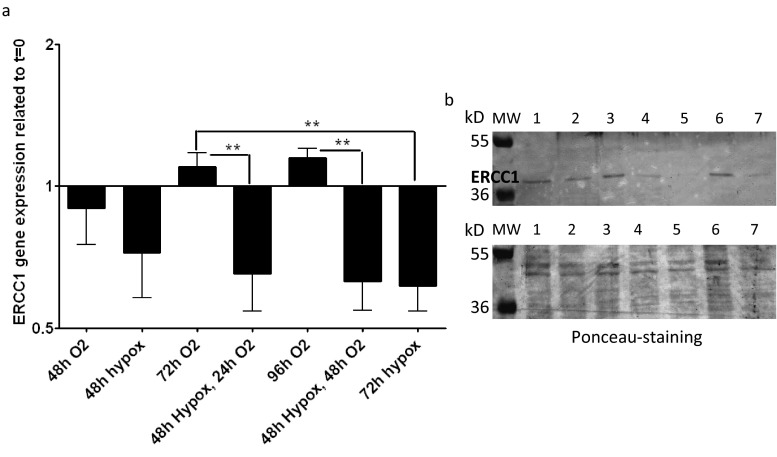
Detroit 562 cells were cultured in hypoxic and normoxic conditions for 48–96 h and ERCC1 mRNA expression was examined (**a**), and western blot of nuclear ERCC1 protein (**b**) was performed. The ERCC1 mRNA expression (**a**) was normalized with β-actin gene expression and was proportioned to the gene expression of control zero (the gene expression of the *t* = 0 days, which was the reference for time and treatment-related changes). “1” represents no change compared to start time (without treatment). The normoxic culture showed no significant fluctuation of ERCC1 gene expression, while the hypoxic culture (even if followed by normoxic) resulted in significant ERCC1 gene expression decrease. Western blot of 8 F1 (**b**, *upper panel*) anti-ERCC1 antibody in cell nuclear extracts of Detroit 562 squamous cell carcinoma cell line cultured 48 h normoxic (*1*), 48 h hypoxic (*2*), 72 h normoxic (*3*), 72 h hypoxic (*4*), 48 h hypoxic and 24 h normoxic (*5*), 96 h normoxic (*6*), and 48 h hypoxic and 48 h normoxic (*7*). Ponceau-stained whole protein detection in nuclear extracts was used as loading control (**b**, *lower panel*). Hypoxic culture conditions, even when followed by normoxic periods, lead to significant reduction of ERCC1 gene expression at mRNA and protein levels

Detroit 562 cells were cultured in hypoxic and normoxic conditions for 48–96 h, and ERCC1 mRNA (Fig. 5a) and cell nuclear protein representation (Fig. 5b) were determined. The ERCC1 mRNA expression (Fig. 5a) was normalized with β-actin gene expression and was related to the gene expression state of the initial 48-h normoxic culture (“control zero”). Supporting the report of Bindra and coworkers [29], 48–72 h hypoxic culture conditions led to a significant reduction of ERCC1 gene expression at mRNA and protein levels compared to normoxic conditions, even when followed by 24–48 h normoxic periods (Fig. 5a, b).

## Discussion

Unexpectedly, 106 HNSCC patients treated with primary concomitant radiochemotherapy responded better, if ERCC1 expression in pretherapeutic tumor samples was high (Fig. 1). This finding was mechanistically elusive at the time of observation, because high ERCC1 expression was considered to indicate low tumor sensitivity to cross-linking chemotherapeutic agents such as CDDP [43] or MMC. This outcome brought us to do some translational experimentation. At first, the specificity of the 8 F1 ERCC1 mouse monoclonal antibody was examined. In immunohistochemical comparisons of tumor tissues and in western blot experiments in tumor cell lines (confirmed by RNA results), 8 F1 yielded specific and plausible results and a relevant bias to low 8 F1 specificity seemed unlikely.

ERCC1 mediates DNA repair by removing therapeutic adducts from tumor DNA. Therefore, the positive clinical effect of high ERCC1 expression in HNSCC patients treated with primary combined radiochemotherapy was unlikely to be related to the chemotherapeutic part. We rather assumed some relation to radiotherapy. It is well known that the effectiveness of radiotherapy on tumor cells is cell cycle dependent [46]. In a next step, we thus examined cell cycle dependency of ERCC1 gene expression. We used an ERCC1-expressing HNSCC cell line (Detroit 562) and a noncancer airway epithelial cell line (BEAS-2B). With these cell lines, we established an ERCC1-immunocytochemical analysis combined with propidium iodide DNA staining for flow cytometry. Using this setup, we could simultaneously assess ERCC1 protein synthesis and cell cycle phase. Actually, in untreated cells, ERCC1 expression was the highest in the G2/M phase (Fig. 4a, e), whose cells are particularly radiosensitive [46]. The association of ERCC1 expression with the radiosensitive cell cycle phase might in part explain the observed clinical benefit of high ERCC1 expression. However, in this context, ERCC1 expression is rather an epiphenomenon, while cell cycle-dependent radiosensitivity is considered an independent underlying cause. However, it is understood that these results in two-cell lines are preliminary and rather have a hypothesis generating than an explanatory character.

The cell cycle relation of ERCC1 function is also discussed by Nunez et al., who were investigating ERCC1-deficient hepatocytes and found that a population of cells arrested in G2 has shown increased DNA content accumulates. This was associated with a p53-independent accumulation of p21. This finding also underlines a G2-related ERCC1 function and a cell-cycle-related ERCC1 expression [4].

We were further interested how chemotherapeutic agents frequently used in the combined treatment of HNSCC affect cell cycle distribution and the relation between cell cycle and ERCC1 expression. To accomplish this, drug concentrations inducing a 50 % growth reduction in comparison to the control untreated cells (IC-50) were established for CDDP, MMC, and 5-FU. The resulting IC-50 values compared well with known data [32–34]. Both CDDP and MMC treatment at IC-50 resulted in shifting cells from the less radiosensitive G1 phase to highly radiosensitive G2/M phase [46], and 20–33 % of cells were detected with fragmented DNA (sub-G1 phase) indicating apoptosis. In contrast, treatment with 5-FU led to S phase arrest or G1-S checkpoint arrest. These data comply with previous studies on radiosensitizing mechanisms of chemotherapeutic agents [48]. Importantly, CDDP treatment induced a significant increase of ERCC1 gene expression in tumor and in normal cells, while MMC and 5-FU did not. However, the relative cell cycle distribution of ERCC1 expression remained unchanged (Fig. 4a, b, e, f). These data support previous studies on radiosensitization by chemotherapeutic agents [48], but again do not provide a mechanistic basis to suggest an independent effect of ERCC1 on treatment outcome in HNSCC patients.

Interestingly, hypoxia reduced ERCC1 gene expression (both at the mRNA and protein levels) and even post-hypoxic, normoxic periods did not recover these changes. In fact, hypoxic cells are up to threefold more resistant to ionizing radiation than cells irradiated under well-oxygenated conditions [49]. Hypoxia appears to be a key microenvironmental factor involved in the development of genetic instability [29], and it also reduces the efficiency of radiation therapy. Bindra et al. reported that mediators of homologous recombination are downregulated by hypoxia [29]. In accordance, our experimental findings in Detroit 562 HNSCC tumor cells also showed that ERCC1 gene expression at the mRNA and protein levels decreased under hypoxia, and even a subsequent normoxic condition did not recover the gene expression changes. Indirectly, it could be assumed that nuclear ERCC1-positive cells in pretreatment biopsy samples might indicate normoxic cells, which are genetically stable, and more radiosensitive than hypoxic cells.

In addition, a recent study highlights that ERCC1 gene expression and protein level does not directly mean functionality. Martens-de Kemp and coworkers from the laboratory of Dr. Brakenhoff recently published that the CDDP-DNA adduct level is the most important determinant of cisplatin sensitivity in HNSCC cells, *which does not correlate* with the gene expression of ERCC1 [50]. These data doubt the direct predictive value of the gene expression level of ERCC1 in pretherapeutic biopsies for the level of CDDP-induced DNA damage and cisplatin effectiveness. Indeed, by immunostaining, we are recognizing gene expression and protein synthesis, which reflect regulatory conditions in the tumor tissue, and the ERCC1 level is an *indicator* of these conditions. The protein function is less mirrored by immunostaining. The current study suggests that ERCC1 staining with mouse monoclonal antibody is an indicator of favorable cell cycle distribution and normoxic conditions.

The current study has several limitations. We analyzed early CR as a marker to treatment response. Identifying early treatment failure moves closer to treatment decision than survival analysis after years. In truth, the follow-up period was rather short in this patient collective for comprehensive survival analysis. Nevertheless, according to Michiels et al. loco-regional control is considered as an effective surrogate endpoint marker [51]. A second limitation is that oropharyngeal carcinomas were overrepresented. Accordingly, Patel et al. have recently published that patients with oropharyngeal HNSCC and high ERCC1 expression were more likely to survive and remain disease-free when compared to nonoropharyngeal squamous cell carcinoma patients with high ERCC1 expression despite treatment modality and human papillomavirus virus (HPV) status [52].

## Conclusion

The results of these investigations suggest that ERCC1 has no predictive value for or against radiochemotherapy in HNSCC on its own, but is an indicator of well-known tumor cell factors as radiosensitive cell cycle phase and normoxic condition, which influence treatment outcome.

## Electronic supplementary material

Below is the link to the electronic supplementary material.

ESM 1 (DOCX 16 kb)

ESM 2 (DOCX 16 kb)

ESM 3 (DOCX 117 kb)

ESM 4 (TIFF 8505 kb)

(GIF 93 kb)

## Abbreviations

5-FU: 5-Fluorouracil
CDDP: Cisplatin, cis-dichloro-diamine-platinum
CT: Computer tomography
DSB: Double strand breaks
ELISA: Enzyme-linked immunosorbent assay
ERCC1: Excision repair cross complementation group 1
FFPE: Formalin-fixed paraffin-embedded
HNSCC: Head and neck squamous cell carcinoma
IC-50: 50 % growth inhibitory (IC-50) concentrations
ICLs: Interstrand DNA cross-links
Ig: Immunoglobulin
MMC: Mitomycin C
MTT: (3-(4,5-Dimethylthiazol-2-yl)-2,5-diphenyltetrazoliumbromide
PBS: Phosphate buffered saline
RCT: Radiochemotherapy
RT-PCR: Reverse transcription-polymerase chain reaction amplification
UPPP: Uvulopalatopharyngoplasties
XPF: Xerodermapigmentosum group F
