# Supplementary material for: Cell cycle association and hypoxia regulation of excision repair cross complementation group 1 protein (ERCC1) in tumor cells of head and neck cancer
Source: Tumour Biol. 2014 May 12;35(8):7807–19. doi: 10.1007/s13277-014-2001-2 (PMC4158184; doi:10.1007/s13277-014-2001-2)
Supplement: Supplementary file 1 — (DOCX 16 kb) [file 13277_2014_2001_MOESM1_ESM.docx]

| Characteristic | n =106 | % |
| --- | --- | --- |
| Sex |  |  |
| Male | 76 | 71.7 |
| Female | 30 | 28.3 |
| Age at diagnosis |  |  |
| Mean | 61.27 |  |
| Range | 29-81 |  |
| Primary tumor site |  |  |
| Oral cavity | 11 | 10.4 |
| Oropharynx | 46 | 43.4 |
| Hypopharynx | 24 | 22.6 |
| Larynx | 16 | 15.1 |
| Others | 9 | 8.5 |
| Clinical UICC stage |  |  |
| I | 0 | 0 |
| II | 2 | 1.9 |
| III | 15 | 14.2 |
| IVa | 74 | 68.8 |
| IVb | 13 | 12.3 |
| IVc | 2 | 1.9 |
| Type of systemic treatment |  |  |
| Radiochemotherapy (CDDP) | 11 | 10.4 |
| Radiochemotherapy (MMC) | 82 | 77.4 |
| Radiochemotherapy (Other) | 2 | 1.9 |
| Radioimmuntherapy (Cetuximab) | 11 | 10.4 |
| Radiation dose |  |  |
| Up to 60 Gy | 6 | 5.7 |
| 60.1-69.9 Gy | 11 | 10.5 |
| 70 Gy | 80 | 76.2 |
| > 70 Gy | 8 | 7.6 |
| Response to treatment |  |  |
| Responder | 68 | 64.2 |
| Non-Responder | 38 | 35.8 |

**Supplementary Table 1. Patient data for therapy response relationship with ERCC1 immunohistochemistry.**
