# Supplementary material for: Cell cycle association and hypoxia regulation of excision repair cross complementation group 1 protein (ERCC1) in tumor cells of head and neck cancer
Source: Tumour Biol. 2014 May 12;35(8):7807–19. doi: 10.1007/s13277-014-2001-2 (PMC4158184; doi:10.1007/s13277-014-2001-2)
Supplement: Supplementary file 2 — (DOCX 16 kb) [file 13277_2014_2001_MOESM2_ESM.docx]

| Characteristic | n =18 | % |
| --- | --- | --- |
| Sex |  |  |
| Male | 9 | 50 |
| Female | 9 | 50 |
| Age at diagnosis |  |  |
| Mean | 60.2 |  |
| Range | 45-73 |  |
| Primary tumor site |  |  |
| Oral cavity | 4 | 22.2 |
| Oropharynx | 9 | 50 |
| Hypopharynx | 1 | 5.6 |
| Larynx | 3 | 16.6 |
| Others | 1 | 5.6 |
| Clinical UICC stage |  |  |
| I | 0 | 0 |
| II | 1 | 5.6 |
| III | 1 | 5.6 |
| IVa | 14 | 77.7 |
| IVb | 2 | 11.1 |
| IVc | 0 | 0 |
| Tumor size |  |  |
| T0 | 1 | 5.6 |
| T1 | 0 | 0 |
| T2 | 3 | 16.6 |
| T3 | 2 | 11.1 |
| T4a | 10 | 55.6 |
| T4b | 2 | 11.1 |
| Nodal stage |  |  |
| N0 | 1 | 5.6 |
| N1 | 1 | 5.6 |
| N2a | 1 | 5.6 |
| N2b | 7 | 38.8 |
| N2c | 8 | 44.4 |

**Supplementary Table 2. Patient data for comparison studies of 8F1, FL297 (ERCC1) and SPM228 (XPF) antibodies.**
