# Supplementary material for: Cell cycle association and hypoxia regulation of excision repair cross complementation group 1 protein (ERCC1) in tumor cells of head and neck cancer
Source: Tumour Biol. 2014 May 12;35(8):7807–19. doi: 10.1007/s13277-014-2001-2 (PMC4158184; doi:10.1007/s13277-014-2001-2)
Supplement: Supplementary file 3 — (DOCX 117 kb) [file 13277_2014_2001_MOESM3_ESM.docx]

Comparison of ERCC1 immunohistochemical representation in oral cavity (n=11), oropharynx (n=46), hypopharynx (n=24) and larynx (n=16). Other localisations contained less than 10 patients.

In oral cavity only 11 patients have been analyzed. The comparison of 5 patients with 6 is statistically not relevant, but RCT non-responder patients show the trend of lower ERCC1 representation.

In oropharynx 46 patients have been analyzed. RCT non-responder patients show the trend of lower ERCC1 representation. Much more patients are responders than non-responders, which makes the comparison unbalanced.

In hypopharynx 24 patients have been analyzed. RCT non-responder patients show highly significant lower ERCC1 representation. In this case the comparison is well balanced, 12 patients are compared with 12 patients. This also means that 50% of RCT-treated hypopharynx HNSCC patients do not respond to the therapy.

In larynx only 16 patients have been analyzed. The comparison of 10 patients with 6 is statistically weak, but here also RCT non-responder patients show the trend of lower ERCC1 representation, however, the difference is not high.
