# Supplementary figures and images for: Cell cycle association and hypoxia regulation of excision repair cross complementation group 1 protein (ERCC1) in tumor cells of head and neck cancer
Source: Tumour Biol. 2014 May 12;35(8):7807–19. doi: 10.1007/s13277-014-2001-2 (PMC4158184; doi:10.1007/s13277-014-2001-2)

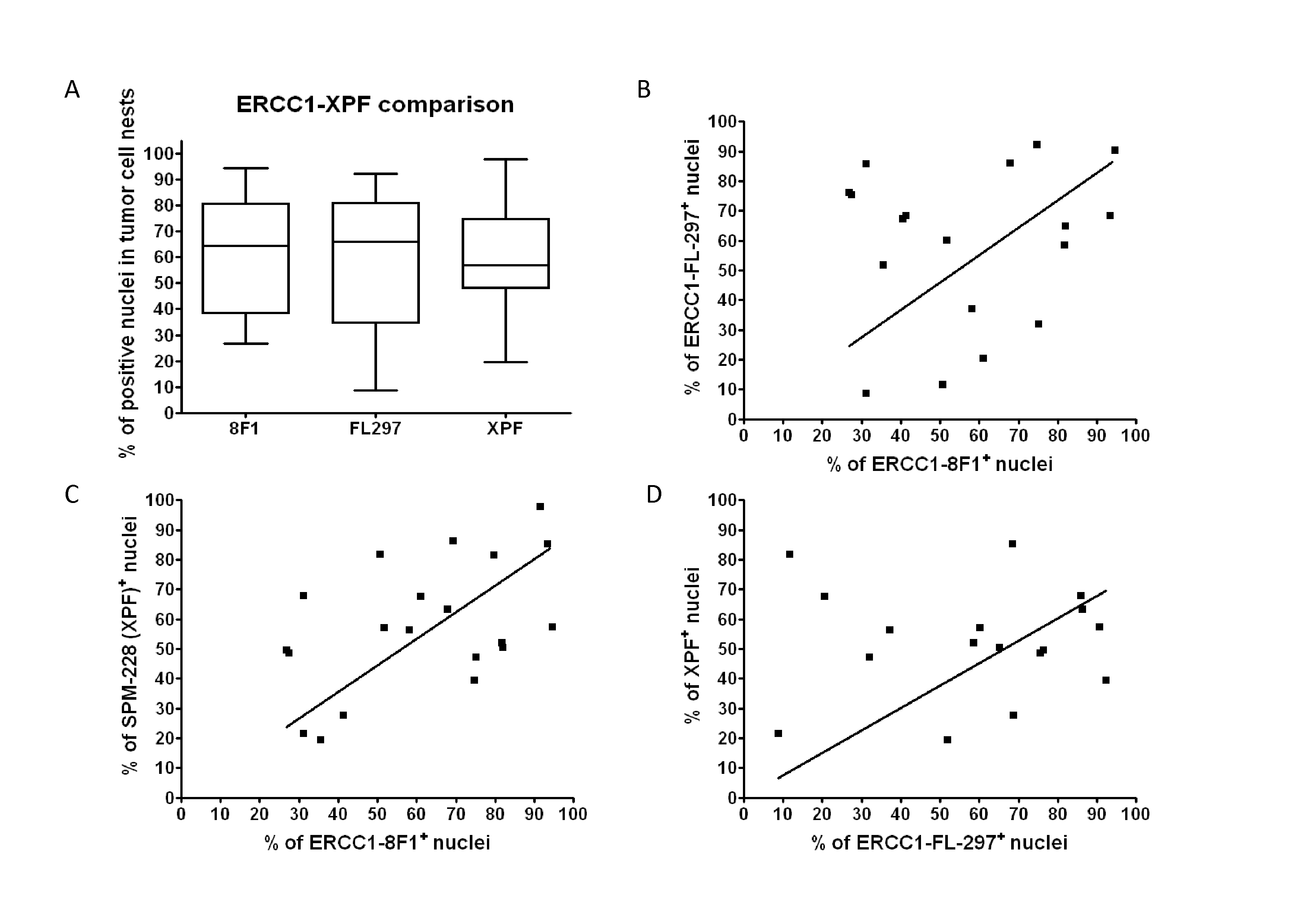

Supplement: Supplementary file 4 — (TIFF 8505 kb) [file 13277_2014_2001_MOESM4_ESM.tif]

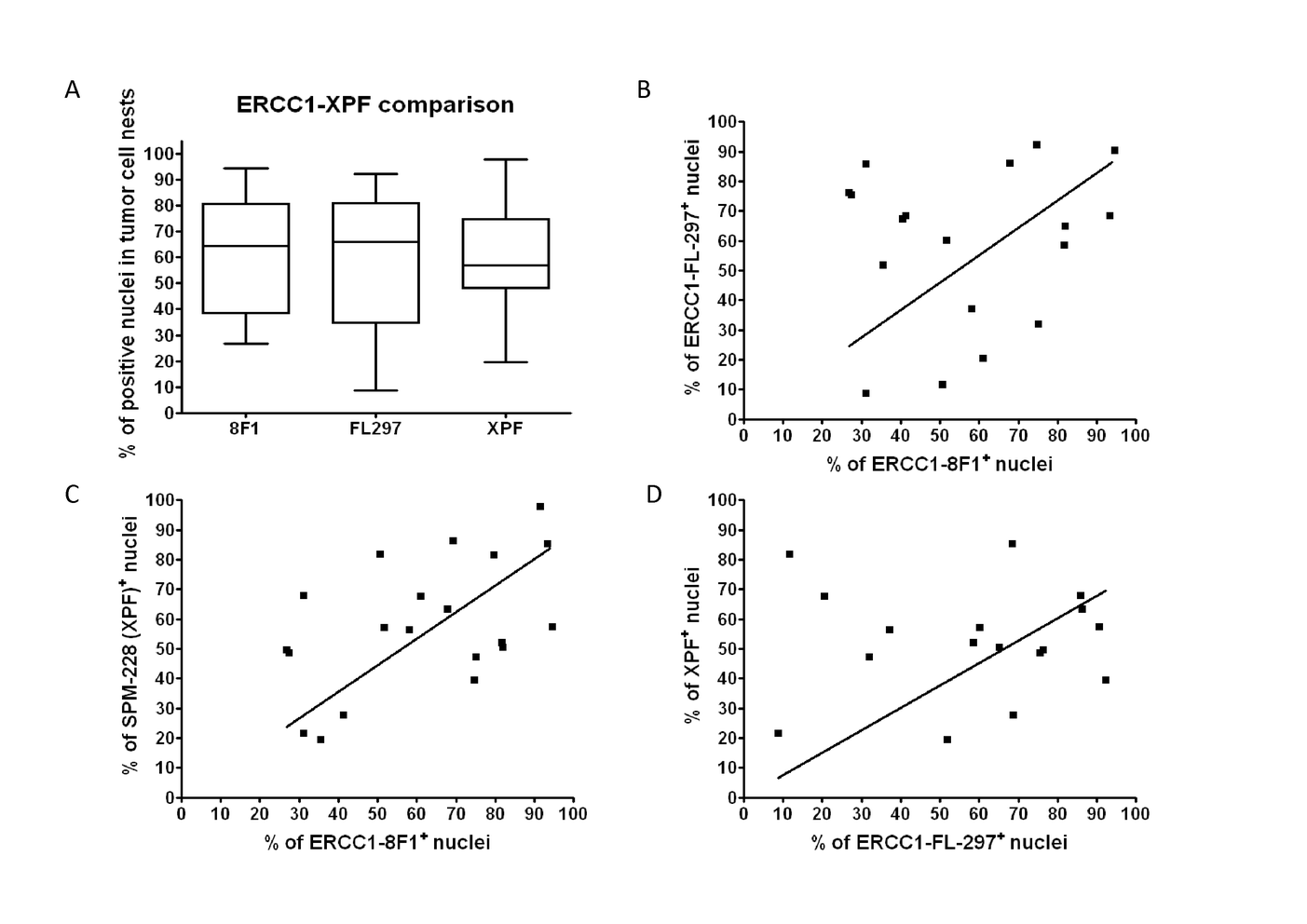

Supplement: Supplementary file 5 — (GIF 93 kb) [file 13277_2014_2001_Fig6_ESM.gif]
